# Step Detection Robust against the Dynamics of Smartphones

**DOI:** 10.3390/s151027230

**Published:** 2015-10-26

**Authors:** Hwan-hee Lee, Suji Choi, Myeong-jin Lee

**Affiliations:** 1 School of Electronics and Information Engineering, Korea Aerospace University, 76 Hanggongdaehang-ro Deogyang-gu, Goyang, Gyeonggi 412-791, Korea; E-Mail: hwanhee4777@gmail.com; 2 Mechatronics R&D Center, Samsung Electronics, 1-1 Samsungjeonja-ro, Hwaseong, Gyeonggi 445-330, Korea; E-Mail: sujiu.choi@samsung.com

**Keywords:** step detection, accelerometer, step average, adaptive magnitude threshold, adaptive temporal threshold, peak-valley relationship, step mode, device pose

## Abstract

A novel algorithm is proposed for robust step detection irrespective of step mode and device pose in smartphone usage environments. The dynamics of smartphones are decoupled into a peak-valley relationship with adaptive magnitude and temporal thresholds. For extracted peaks and valleys in the magnitude of acceleration, a step is defined as consisting of a peak and its adjacent valley. Adaptive magnitude thresholds consisting of step average and step deviation are applied to suppress pseudo peaks or valleys that mostly occur during the transition among step modes or device poses. Adaptive temporal thresholds are applied to time intervals between peaks or valleys to consider the time-varying pace of human walking or running for the correct selection of peaks or valleys. From the experimental results, it can be seen that the proposed step detection algorithm shows more than 98.6% average accuracy for any combination of step mode and device pose and outperforms state-of-the-art algorithms.

## 1. Introduction

Billions of smartphones are in use around the world, mainly for voice, visual and data communications. Most smartphones are equipped with inertial sensors composed of accelerometers, gyroscopes and magnetometers. Inertial sensors have been used in various applications, such as navigation for aerial and land vehicles, robotics, *etc*. The rapid development of micro-electro-mechanical systems (MEMS) has presented new possible application areas of inertial sensors, such as human activity monitoring [1,2,3,4,5], fall detection [5,6], medical treatment [7], remote rehabilitation and physical therapy [8,9,10,11,12], sports [13,14], education [15], security [16], life logging [17,18], *etc*. Pedestrian dead reckoning (PDR) or indoor/outdoor localization are important application areas for these sensors. PDR approaches are based on detecting steps and step headings to estimate the current user position. Recent research has shown that the accuracy of PDR for low-cost inertial sensors used by smartphones can make these devices an affordable way to realize localization of pedestrian users [18,19,20,21,22].

These applications commonly utilize an accelerometer to obtain users’ motion information, such as their gait, physical activity or displacement. Although these applications are different from each other in their output forms, *i.e*., step count [23,24,25,26,27,28,29], step mode [11,12,19,22], energy expenditure [4,5,12] and displacement or speed [5,8,21,22,30,31], most of them detect user’s steps, which can serve as fundamental information for output estimation.

Conventional studies of step detection based on accelerometers have used threshold-based [16,26,27,28,29,32,33,34], peak detection [18,21,22,23,24,25,30,35], auto-correlation [19], or spectral analysis [17]. Most step detection methods have assumed that sensors are tied to pre-fixed positions [23,24,25,26,27,28] and have also assumed specific step modes, such as walking, running or static, for the configuration of their algorithms [24,25,26,27,29]. Since smartphones are typically carried by users in a free manner rather than being tied to pre-fixed positions on the human body, and because human walking speed varies over time, step detection research for smartphones is very challenging. Some studies have tried to consider the dynamics of smartphones by explicit estimation of device pose [22,23,29] or step mode [22,26,28] or by using adaptive thresholds for peak validation without estimating the step mode or device pose [18,35]. However, these algorithms cannot handle with acceptable accuracy cases in which step mode and device pose are continuously changing.

In this paper, for robust step detection in real smartphone usage environments in which step mode and device pose are continuously changing, a novel step detection algorithm is proposed. To suppress noisy peaks or valleys, without estimating step mode or device pose, all of the variabilities in the magnitude of acceleration for every combination of step mode and device pose are integrated into stable measures reflecting the short-term and long-term variation of such factors as step average, step deviation and the statistics of time intervals between peaks or valleys. To validate peak and valley candidates in the magnitude and temporal directions, these measures are used for the calculation of adaptive thresholds.

The contributions of this paper are three-fold. First, the dynamics of smartphones can be decoupled into a peak-valley relationship without estimating step mode and device pose. Second, to consider the short-term and long-term variation in the magnitude of acceleration into step detection, adaptive magnitude and temporal thresholds are proposed, which are calculated from step averages, step deviations and the statistics of time intervals between peaks or valleys. Third, the proposed step detection algorithm can provide an acceptable level of step detection accuracy for any combination of step mode and device pose and for a wide range of sampling rates of acceleration.

The organization of this paper is as follows. In Section 2, related works on step detection are reviewed. In Section 3, statistics of magnitude of acceleration obtained from the dynamics of smartphones are analyzed for various step detection environments. In Section 4, a step detection algorithm is proposed that decouples the dynamics of smartphones into the peak-valley relationship using adaptive magnitude and temporal thresholds. In Section 5, the experimental results of the proposed algorithm are presented and compared to those of state-of-the-art step detection algorithms. In Section 6, conclusions are presented.

## 2. Related Work

Conventional studies on step detection using accelerometers are based on thresholds [16,26,27,28,29,32,33,34] peak detection [18,21,22,23,24,25,30,35], auto-correlation [19] or spectral analysis [17].

Threshold-based approaches include simple threshold [26,27,28,29,33,34] and zero velocity update (ZUPT) methods [16,32]. Simple threshold methods compare the magnitude of acceleration, its low-pass filtered version or other measures based on acceleration with pre-configured thresholds. ZUPT methods utilize the fact that each foot is regularly static during normal walking motion and that the accelerometer must report a certain period of inactivity [16,32]. Conventional ZUPT methods can be successful for step detection when the accelerometer is mounted on a foot [16,32]. However, most threshold-based methods in which accelerometers are placed inside smartphones do not achieve good performance in general smartphone usage environments in which smartphones are typically carried freely by users rather than being held in a pre-fixed position [26,27,28,29,33,34].

Peak detection-based approaches also utilize the periodic characteristics of the magnitude of acceleration caused by the repetitive motion of walking or running [18,21,22,23,24,30,35]. All of the peak detection methods extract local peaks in the magnitude of acceleration and count a step for each peak. To reduce over-counting from invalid extracted local peaks, each peak is validated in the magnitude or temporal direction by thresholds for the magnitude of acceleration [18,21,24,35], by the temporal constraint from the feasible walking speed [18,21,22] or by the vertical displacement constraint [30]. Although peak detection-based methods are low in complexity, they are limited to special environments of certain step modes and device poses. Their performance may degrade during a transition of the step mode or device pose in smartphone-based applications.

Spectral analysis [17] and auto-correlation-based approaches [19] utilize the periodic characteristics of the magnitude of acceleration in the spectral or time domains by using transformations, such as the discrete Fourier transform (DFT) or dynamic time warping (DTW), or by using auto-correlation. However, these approaches are limited in their application to mobile environments due to their large computational loads.

Since smartphones are typically carried freely by users, step detection research for smartphones is very challenging. A smartphone user can make a phone call, send a text message while watching the display, swing the smartphone in his or her hand or put it in a pocket of his or her pants, or in a backpack, or a handbag, or in an arm-band, while he or she is in motion. This kind of smartphone usage is defined in this study as device pose. Most conventional step detection methods have difficulty handling device pose, because they assume that the sensors are set in pre-fixed positions [23,24,25,26,27,28]. Because these methods also have difficulty handling the step mode, especially in determining transitions among step modes, a large number of conventional methods also assumed a specific step mode, such as walking, running or static, for the configuration of their algorithms [24,25,26,27,29].

Adaptive thresholds for the magnitude of acceleration have been used in conventional studies for accurate step detection [18,21,22,26,35,36]. The authors in [18,21,35] tried to consider the time-varying step mode for peak detection by calculating the threshold adaptively using window-based statistics of acceleration. Although these algorithms may improve the accuracy of step detection for larger windows in the same or similar step modes, larger windows may cause steps to be missed during the transition of the step mode or the device pose.

To tackle these difficulties in configuring appropriate parameters for step detection considering step mode and device pose, threshold-based estimation of step mode [26,28], threshold-based estimation of device pose [29] and machine learning-based classification of step mode [23] have been considered. Furthermore, for indoor localization applications in which acceleration is used for step detection or for similar purposes, machine learning-based classification of step mode or device pose has been proposed [22,31]. Although there exists one study performed by Zhang *et al*. that attempted to estimate both step mode and device pose by classification, the classification performance was less than 80% for the running mode, which means that further improvement is required for accurate step detection or indoor localization [22].

For step detection with unrestricted device pose, Pan *et al*. proposed a step counting algorithm allowing arbitrary device pose while walking [37]. After deriving horizontal components of linear acceleration vectors from device pose transformation based on gravity vectors, possible correlated segments are identified from the gathered raw data and taken as user’s steps. Although the step detection error is quite small for various device poses and dynamically-changing carrying means, the authors did not consider the dynamics of step mode, but limited their algorithm to walking mode. Brajdic *et al*. evaluated the performance of conventional walk detection and step counting algorithms in unconstrained smartphones, with device pose fixed to any among possible poses and with varying walking speed [38]. Although the authors supported threshold-based algorithms in accuracy and computational complexities, they did not evaluate threshold-based algorithms for more realistic conditions, such as running and device pose change during running or walking, to show the limitations explained above.

The dynamics of smartphones from various combinations of step mode and device pose may be difficult to estimate explicitly for step detection. Although there may exist differences in sampled acceleration among certain distinctive step modes or device poses, the authors argue that the dynamics of smartphones cannot be handled by simply assigning an explicit step mode or device pose, but should be treated using a unified algorithm that can extract the characteristics of steps that are invariant over the dynamics.

## 3. Dynamics of Acceleration in Real Smartphone Usage Environments

There have been several arguments to describe the aspects of real smartphone usage environments that affect the performance of step detection. The arguments include the step mode and the device pose of the user. The step mode varies over time during the user’s moving period and may fall into either walking or running mode. In each step mode, each peak in the magnitude of acceleration can be a candidate for a step. While most of the peaks can be determined as real steps, there exist pseudo peaks that are very close in time to the peaks determined as steps, but that are not real peaks. To suppress these pseudo peaks that do not contribute to steps, peak detection algorithms with window-based thresholds have been proposed [18,23].

**Figure 1:**
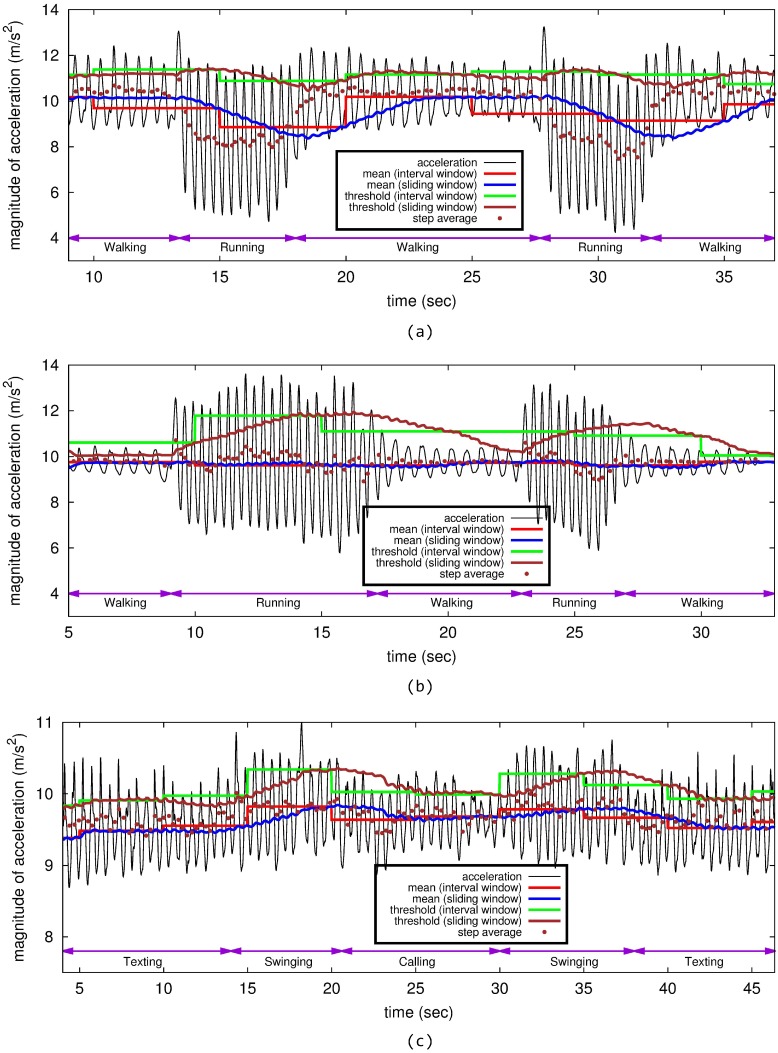
Time-varying statistics of the magnitude of acceleration during transitions of step mode or device pose. (**a**) Missing peaks during step mode transitions: walking to running; (**b**) missing peaks during step mode transitions: running to walking; (**c**) missing peaks during device pose transition: walking.

Figure 1a,b shows example traces of the magnitude of acceleration, means and thresholds based on the mean and the standard deviation proposed by Chon *et al*. [18] and Susi *et al*. [23] for time-varying step mode with device pose fixed. The sizes of the interval and sliding windows are all five seconds. In each step mode, the walking speed may vary over time, and the distribution of the magnitude changes over time; especially, the variance of the magnitude in running mode is larger than that in walking mode. The average time interval between adjacent peaks differs across step modes: the interval in running mode is shorter than that in walking mode. The mean in each step mode, which can be calculated using either interval or sliding window-based method, for either running or walking, does not change abruptly. However, the mean right after the step mode transition is quite different from its previous value before the transition.

Although interval- or sliding window-based statistics may provide quite accurate thresholds for peak detection for constant step mode and device pose, these thresholds sometimes fail to detect peaks when the step mode or the device pose changes abruptly. For both algorithms [18,23], there are six missing peaks among 62 peaks and 20 missing peaks among 60 peaks during step mode transitions from walking to running, as shown in Figure 1a, and from running to walking, as shown in Figure 1b, respectively. This is because the means in these algorithms cannot be the middle positions, which are normally suitable for peak detection; also, the standard deviation cannot adapt to the time-varying statistics of acceleration quickly enough.

Although abrupt change in the mean or standard deviation is not always found for time-varying device pose with step mode fixed to walking, as shown in Figure 1c, gradual changes in the statistics can also affect the step detection accuracy due to the window-based threshold calculation. Furthermore, the statistics for each device pose are similar to those of other poses, except for pocket and backpack, as shown in Table 1. Free-walking is a step mode in which each participant changes his or her walking speed arbitrarily in time; this mode emulates the transition of step mode between walking and running.

These observations show that the step detection algorithm for time-varying step mode and device pose should be designed not by simply applying statistics for peak detection, but by using other features of steps that are robust against changes of step mode or device pose. For time-varying step mode and device pose shown in Figure 1, a peak-valley pair can be easily differentiated, even for the transitions of step mode and device pose. The authors argue that the statistics of peaks and valleys in the magnitude and temporal domains can reflect the time-varying statistics of the magnitude of acceleration for the transitions of step mode or device pose.

**Table 1:** Statistics of the magnitude of acceleration in various smartphone usage environments (m/s${}^{2}$).

Step Mode		Device Pose						
		*Texting*	*Swinging*	*Calling*	*Pocket*	*Backpack*	*Handbag*	*Arm-Band*
walking	mean	9.485	9.790	9.679	10.254	10.172	9.512	9.773
	SD	0.426	0.489	0.386	1.251	0.709	0.480	0.486
running	mean	9.460	9.754	9.545	9.278	8.641	9.838	9.079
	SD	1.064	1.358	1.685	2.293	2.115	1.258	2.338
free-walking	mean	9.469	9.741	9.596	9.882	9.612	9.808	9.555
	SD	0.972	1.151	1.182	1.868	1.819	1.074	1.683

## 4. Proposed Step Detection Algorithm Robust against Step Mode and Device Pose

**Figure 2:**
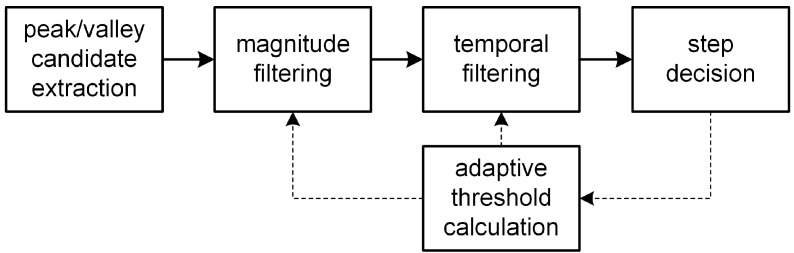
Proposed step detection algorithm.

In this section, a novel step detection algorithm is proposed that can adapt to the time-varying magnitude of acceleration and the pace of steps caused by the time-varying step mode and device pose of a smartphone user. The proposed step detection algorithm is shown in Figure 2; the algorithm consists of peak and valley candidate extraction, magnitude filtering, temporal filtering, step decision and adaptive threshold calculation for magnitude and temporal filtering.

### 4.1. Peak and Valley Candidate Detection with Adaptive Magnitude Thresholds

To overcome the limitations of conventional studies on adaptive threshold-based step detection [18,21,35], validation of peaks and valleys is performed after detecting peak and valley candidates using adaptive thresholds that reflect time-varying step mode and device pose.

For fixed step mode and device pose, there still exists variation in the peak magnitude. The variation in the peak magnitude gets larger for faster human walking speeds, as shown in Table 1. Chon *et al*. tried to determine this variation using window-based threshold calculation; they obtained an acceptable level of accuracy for a large window size [18]. The threshold consists of the average and the standard deviation of the magnitude of acceleration in a fixed window. However, increasing the window size may degrade the step detection accuracy during the transition of step mode or device pose, because the threshold calculated from a larger window may not be able to effectively handle the variation in the recent statistics.

To reflect changes in step mode and device pose for step detection in real time, the step average is defined as follows.

(1) $$\begin{array}{c} \mu_{a}=\frac{|a_{p}\left|+\right|a_{v}|}{2} \end{array}$$

where $a_{p}$ and $a_{v}$ represent the accelerations of the peak and the valley detected most recently, respectively. This is the average magnitude of the recent peak-valley pair, *i.e*., the peak and its adjacent valley, and is updated for every peak or valley detected. Step deviation is defined as the standard deviation of the magnitude of acceleration for recent *K* acceleration samples.

The step average is proposed to be used to catch the short-term variation in the statistics of acceleration, such as the transition of step mode and device pose. Furthermore, step deviation is proposed to determine the long-term variation in the statistics of acceleration with the same step mode and device pose. Because the step average can reflect the time-varying statistics of acceleration for any combination of step mode and device pose, the average can quickly adapt to transitions of step mode or device pose. For constant step mode and device pose, the step average is similar to that in the previous step. However, if the step mode or device pose changes, the step average may become quite different from its previous value.

Based on he observations of the step average, the type of *n*-th acceleration sample $a_{n}$ is determined using adaptive magnitude thresholds, as follows.

(2) $$\begin{array}{c} t_{a}=\left\{\begin{array}{cc} peakcandidate, & |a_{n}\left|>\max(\right|a_{n-1}\left|,\right|a_{n+1}\left|,\mu_{a}+\frac{\sigma_{a}}{\alpha }\right)\\ valleycandidate, & |a_{n}\left|<\min(\right|a_{n-1}\left|,\right|a_{n+1}\left|,\mu_{a}-\frac{\sigma_{a}}{\alpha }\right)\\ intermediatesample, & otherwise \end{array}\right. \end{array}$$

where $\mu_{a}$, $\sigma_{a}$ and *α* represent the step average, the step deviation and a magnitude constant, respectively.

**Figure 3:**
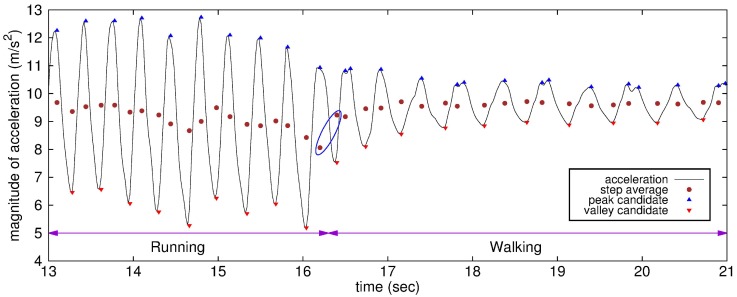
Step mode transition.

Figure 3 shows the step average and the detection results for peak and valley candidates determined using the proposed algorithm for step mode transition from running to walking. The step average reflects the magnitudes of incoming acceleration samples in real time and, as such, is updated dynamically. Because every step average is always located in the middle of a peak and its adjacent valley, it can be used as a reference magnitude for peak-valley detection. The magnitude of the first valley right after a transition is largely increased compared to that of its adjacent valley before the transition. The same applies to the step average after the transition.

### 4.2. Validation of Peak and Valley Candidates with Adaptive Temporal Thresholds

**Figure 4:**
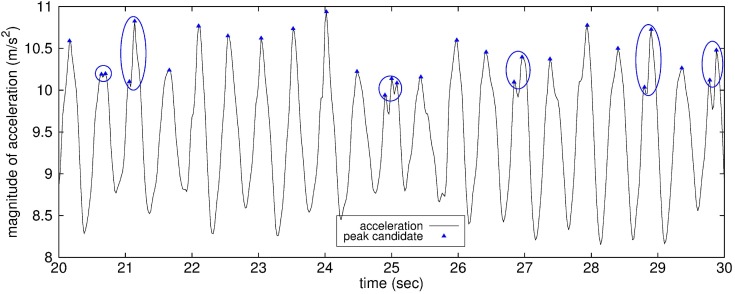
Group of peak candidates gathered in a very short time range.

Some peak or valley candidates still gather in certain short time ranges of which the lengths are much less than the average step interval. Figure 4 shows several peak candidates gathered in very short time intervals in walking mode. This gathering of the peak candidates may be caused by some irregularity of human behavior, especially in walking mode; it may be due to walking in a halting way or to changing the walking direction.

For each group of peak candidates gathered in a certain short time range, accurate step detection requires that only one peak candidate be selected as the real one. For this purpose, conventional studies have proposed to exclude peak candidates outside a pre-defined time range after each peak occurrence [18,21]. This time range is set according to the possible walking pace in each step mode: 0.2–2.0 s [21] or 0.2–1.5 s [18] after each peak. Although this idea may be effective for fast step mode, pseudo peaks existing between real peaks in slow step mode cannot always be suppressed using the fixed time range if the time range does not reflect the speed of walking or the step interval.

In this study, to eliminate from step counting peak candidates that are quite close in time to the real peaks or to extract only one peak from a gathered group of peak candidates in a very short time range, every peak candidate is validated by checking the time distance to the recent peak using the following threshold.

(3) $$\begin{array}{c} Th_{p}=\mu_{p}-\frac{\sigma_{p}}{\beta } \end{array}$$

where $\mu_{p}$, $\sigma_{p}$ and *β* represent the average and the standard deviation of the time interval between adjacent peaks in the magnitude of acceleration and a time scale constant, respectively.

To extract only one valley from a gathered group of valley candidates in a very short time range, every valley candidate is validated by checking the time distance to the recent valley using the following threshold.

(4) $$\begin{array}{c} Th_{v}=\mu_{v}-\frac{\sigma_{v}}{\beta } \end{array}$$

where $\mu_{v}$ and $\sigma_{v}$ represent the average and the standard deviation of the time interval between adjacent valleys in the magnitude of acceleration, respectively. These averages and the standard deviations are calculated for recent *M* peaks or valleys.

### 4.3. Proposed Step Detection Algorithm Based on Adaptive Thresholds

The proposed step detection algorithm is described using the notations in Table 2 as in Algorithm 1, which utilizes the functions in Algorithms 2, 3 and 4. Algorithm 2 describes the detection of peak and valley candidates for incoming acceleration samples. The type of the acceleration sample $a_{n}$ ($S_{c}$) is determined using the adaptive magnitude thresholds in Equation (2) and returned. Algorithm 1 is executed for every incoming acceleration sample. It determines the state of $a_{n}$ ($S_{n}$) and current step count from the inputs consisting of the state of $a_{n-1}$ ($S_{n-1}$), recent step count (count) and $a_{n+1}$. During the initial startup of the algorithm execution, only a peak candidate can be determined to be a new peak as in Line (1), and any valley candidate is discarded. If a peak candidate found in the valley state ($S_{valley}$) satisfies the adaptive threshold for the peak interval, it is determined to be a new peak, and the parameters related to peaks are updated as in Line (2). If a peak candidate in the peak state ($S_{peak}$) is close in time to the recent peak and its magnitude is larger than that of the recent peak, it replaces the recent peak as in Line (3). If a valley candidate in the peak state ($S_{peak}$) satisfies the adaptive threshold for the valley interval, it is determined to be a new valley, and the step counter is increased by one as in Line (4). If a valley candidate in the valley state ($S_{valley}$) is close in time to the recent valley and its magnitude is smaller than that of the recent valley, it replaces the recent valley as in Line (5).

Algorithms 3 and 4 describe the processes after finding peaks or valleys. The statistics of the time interval between adjacent peaks (or valleys), the occurrence time and the magnitude of acceleration are updated for every peak or valley found. The step average $\mu_{a}$ is updated for the first peak or valley right after the recent valley or peak as in Line (2) or (4) of Algorithm 1, respectively.

**Table 2:** Notations.

$a_{n}$	3-dimensional acceleration vector at sample time *n*
$a_{p}$	3-dimensional acceleration vector of the recent peak
$a_{v}$	3-dimensional acceleration vector of the recent valley
|a|	the magnitude of acceleration of vector $a^{*}$
$\mu_{a}$	the step average defined in Equation (1)
$\mu_{p}$	the average time interval between adjacent peaks
$\mu_{v}$	the average time interval between adjacent valleys
$\sigma_{a}$	the step deviation of the magnitude of acceleration
$\sigma_{p}$	the standard deviation of the time interval between adjacent peaks
$\sigma_{v}$	the standard deviation of the time interval between adjacent valleys
$t_{a_{n}}$	the type of the acceleration sample $a_{n}$
$Th_{p}$	the adaptive time threshold for peaks
$Th_{v}$	the adaptive time threshold for valleys

* $\left|a|=\right|\left(a_{x},a_{y},a_{z}\right)|=\sqrt{a_{x}^{2}+a_{y}^{2}+a_{z}^{2}}$.


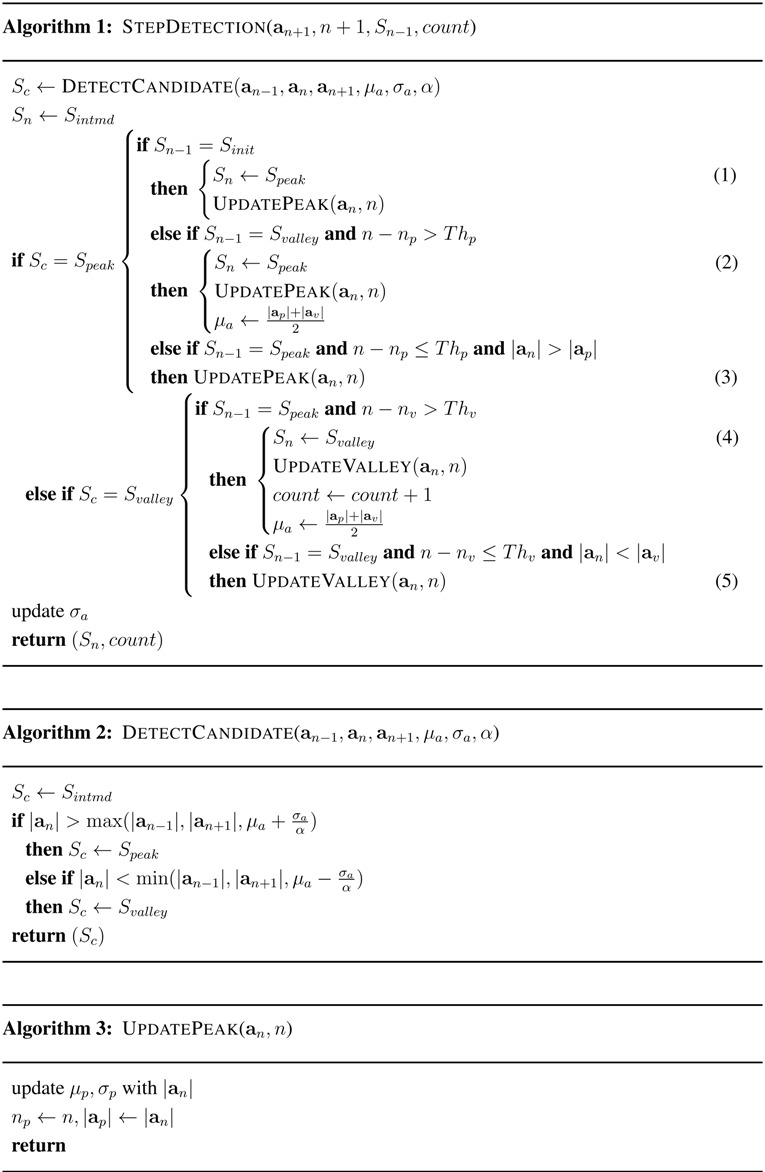


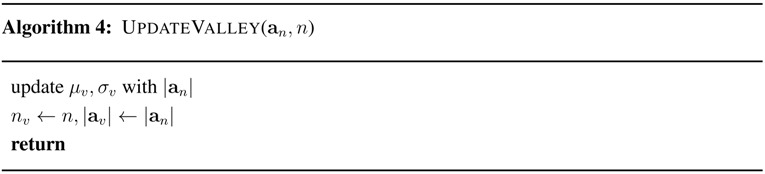


## 5. Experimental Results

### 5.1. Experimental Environments

The proposed step detection algorithm is implemented on Android smartphone platforms. The experiments consist of three parts. The first part is an evaluation of the accuracy of step detection for every combination of step mode and device pose. The second part is a comparison of the performance of the proposed algorithm to those of state-of-the-art algorithms for each step mode with fixed device pose and for time-varying device pose with fixed step mode. The third part is an investigation of the accuracy of step detection and of the power consumption with respect to the sampling rate of acceleration. The sampling rate of acceleration was set at 50 Hz, except in the third experiment.

The influence of parameters *K*, *M*, *α* and *β* on the step detection accuracy is investigated using the acceleration data collected in every combination of step mode and device pose. Parameter *K* should be selected such that the step deviation can reflect the long-term variation in the statistics of acceleration. Parameter *α* should be assigned so as not to disturb the peak or valley detection due to large step deviation during step mode change, especially from running to walking. Parameters *M* and *β* should be configured such that the statistics of peak or valley intervals can reflect the time-varying speed of walking or running and the noisy peaks or valleys can be delineated from real peaks or valleys. From our observation, *K* does not affect the step detection accuracy in any step mode and device pose if the sampling rate is larger than 10 Hz. The value of 25 is assigned to *K* to cover one step cycle in normal walking speed with the sampling rate of 50 Hz. The step detection accuracy saturates to its maximum for any step mode and device pose if *α* and *M* are larger than four and 10 and if *β* is less than 0.4, respectively. Irrespective of the step mode, device pose and sampling rate, values of 25, 10, 4 and $\frac{1}{3}$ were assigned to *K*, *M*, *α* and *β*, respectively.

**Figure 5:**
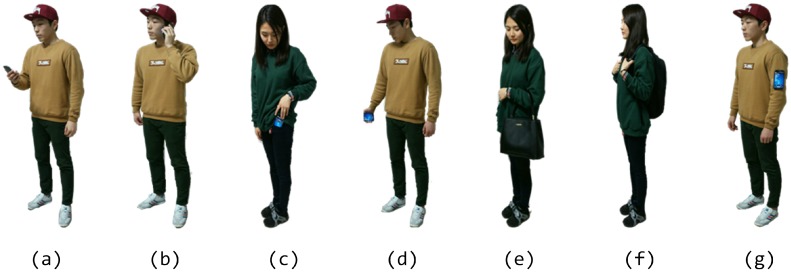
Device pose (**a**) texting; (**b**) calling; (**c**) pocket; (**d**) swinging; (**e**) handbag; (**f**) backpack; (**g**) arm-band.

For performance evaluation of the proposed algorithm with various combinations of step mode and device pose, seven smartphones, including at least one Galaxy S3, Galaxy S4 and Vega LTE-A, were used for the experiment, all with Android platforms. Fifteen men participated in the experiment; their heights were from 170–184 cm, and their ages were from 18–28. The same number of women also participated in the experiment; their heights were from 159–171 cm, and their ages were from 17–26. Three step modes, walking, running and free-walking, and seven device poses, shown in Figure 5, were used for the experiment. The speeds of walking and running by the participants fell into 1.6–2.1 and 2.2–3.5 steps/s, respectively. Step detection was performed for each participant for every combination of step mode and device pose. Each participant made 300 steps in each combination of step mode and device pose; the steps counted by the proposed algorithm are recorded for performance evaluation.

For a performance comparison between the proposed algorithm and state-of-the-art algorithms, two commercial algorithms embedded in smartphones [39,40] and one algorithm from the literature [18] were used. Several combinations of step mode and device pose, which happen frequently in real smartphone usage environments, were tried for a reduced set of participants of five men and five women. To compare the performance for more realistic smartphone usage environments, time-varying device pose in walking step mode was also used for the experiment.

Finally, the accuracy of the step detection and the corresponding power consumption with respect to the sampling rate of acceleration were investigated. For every configurable sampling rate in the smartphone used, Galaxy S4, the power consumption was measured using a Monsoon power meter [41] for 150 s, which roughly corresponds to 300 steps in walking step mode. The accuracy was measured for 300 steps in free-walking step mode and in texting device pose, with every configurable sampling rate, for five men and five women.

### 5.2. Performance Evaluation with Fixed Step Mode and Device Pose

Table 3 shows the performance of the proposed step detection algorithm for every combination of step mode and device pose. Each number represents the average accuracy obtained by fifteen men and fifteen women for each combination of step mode and device pose. The experimental results show that the performance of the proposed algorithm is not greatly affected by any specific step mode or device pose, but maintains a high level of accuracy consistently over any combination of step mode and device pose. Because free-walking mode allows the walking speed to change arbitrarily over time, it can also be argued that the proposed algorithm can detect steps at 99.3% of the overall accuracy with fixed device pose, irrespective of step mode.

**Table 3:** Average accuracy of the proposed step detection algorithm.

Device Pose		Step Mode		
		Walking	Running	Free-Walking
texting	men	99.5	99.6	99.4
	women	99.6	99.3	99.0
swinging	men	99.5	99.5	99.5
	women	99.6	99.2	99.3
calling	men	99.9	99.6	99.5
	women	99.7	99.5	99.4
pocket	men	99.6	99.4	99.0
	women	99.0	99.3	99.0
backpack	men	99.6	99.5	99.5
	women	99.7	99.4	99.5
handbag	men	99.6	99.3	99.3
	women	99.5	98.6	99.0
arm-band	men	99.7	99.9	99.7
	women	99.7	99.8	99.8
overall	men	99.6	99.5	99.4
	women	99.5	99.3	99.3
	all	99.6	99.4	99.3

### 5.3. Performance Comparison with State-of-the-Art Algorithms

The performance of the proposed algorithm was compared to those of two commercialized algorithms and one algorithm from the literature: S-Health on Galaxy S4 [39], Health on iPhone 5s (i-Health) [40] and Chon’s algorithm in [18].

#### 5.3.1. Fixed Device Pose

**Figure 6:**
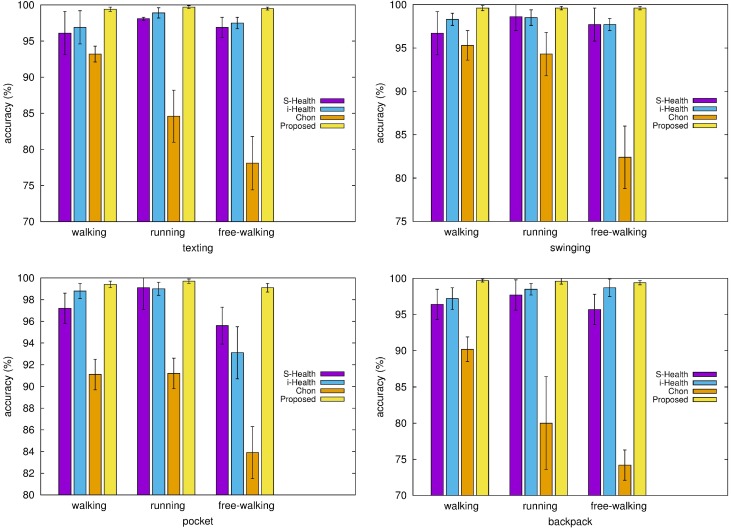
Performance comparison of step detection algorithms.

Figure 6 shows the average and the standard deviation of the step detection accuracy for all possible combinations of the three step modes and the four device poses. All of the algorithms showed more than 90.2% accuracy in walking step mode with various device poses. Three algorithms, S-Health, i-Health and the proposed one, showed consistent and acceptable levels of accuracy in every combination of step mode and device pose. For S-Health and i-Health, the accuracy in running is higher, and the variation is lower than those of the other step modes. This is because the range of the magnitude of acceleration in running is larger than those in the other step modes; this fact allows the algorithms to detect steps easily. However, in the proposed algorithm, there is no such performance enhancement in running mode, because the algorithm has already reached high enough accuracy in every combination of step mode and device pose. Among the step detection algorithms, the proposed algorithm shows the highest accuracy and the lowest variation in accuracy. Chon’s algorithm shows a lower accuracy and larger variability of accuracy than the other algorithms.

The proposed algorithm shows a performance of more than 99.4% for every step mode; the variability of the accuracy over different step modes is quite small. This is because the proposed step detection algorithm uses adaptive magnitude and temporal thresholds from the statistics of peak-valley pairs; these pairs can be differentiated easily from each other, even for the transition of step mode and device pose, and can reflect the time-varying statistics of acceleration.

#### 5.3.2. Time-Varying Device Pose

To evaluate the performance for time-varying device poses, each participant walked 300 steps in walking step mode while repeating the device pose transition in the order of texting, swinging and calling. These three device poses are selected because they happen most frequently in the real environment. The other device poses are not considered because device pose transitions to or from them are not feasible or are less frequent.

Table 4 compares the performances for the time-varying device pose in walking step mode. The proposed algorithm shows an average accuracy of 98.7%, which is lower by only 0.8% from the average accuracy for fixed device pose in walking step mode, shown in Figure 6. The other algorithms showed more than 2.2%–22.6% performance degradation for the time-varying device pose.

**Table 4:** Performance for time-varying device pose in walking step mode.

Algorithm	Accuracy (%)	
	Average	Degradation
S-Health	94.4	2.2
i-Health	82.3	15.5
Chon [18]	69.9	22.6
Proposed	98.7	0.8

### 5.4. Cost of Step Detection: Power Consumption and Accuracy

From the evaluation results by Brajdic *et al*., a window-based peak detection is a cost-effective option for step counting regardless of smartphone placement [38]. The calculation cost of the proposed step detection algorithm is compared to that of Chon’s window-based peak detection algorithm with adaptive thresholds [18] by using the method profiler of Android Dalvik Debug Monitor Server (DDMS) on Galaxy S4. The proposed algorithm is shown to consume 20% less CPU time than Chon’s algorithm.

**Figure 7:**
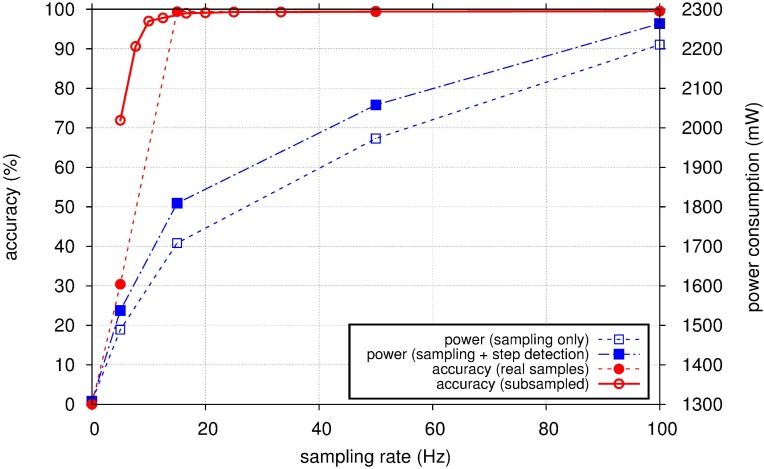
Power consumption and accuracy of step detection with respect to the sampling rate.

Because the cost for step detection in smartphones is closely related to power consumption, two experiments were performed for the investigation of the accuracy and of the power consumption from acceleration sampling and the execution of the proposed algorithm with respect to the sampling rate. The first measured the sampling power consumption, the overall power consumption and the corresponding accuracy for configurable sampling rates. The smartphone used for the experiment has a limitation in configuring the sampling rate; it can only set values of 100, 50, 15 and 5 Hz. Figure 7 shows the power consumption and the accuracy for the configurable sampling rates. The power consumption monotonically increases from static power consumption at zero sampling rate as the sampling rate increases. The sampling power consumption contributes to the overall power consumption greatly. The power consumption for the algorithm execution is less than 11% of the overall power consumption. The accuracy of step detection also increases for increasing sampling rates, exceeding 99.3% for sampling rates over 15 Hz.

The second measured the accuracy of the proposed algorithm for a sampling rate subsampled from the maximum configurable sampling rate of 100 Hz. The purpose of this experiment was to find the minimum acceptable sampling rate for the proposed algorithm that could be used in other smartphone devices for lower power consumption. The proposed algorithm is shown to have an accuracy exceeding 90% at a sampling rate of over 8 Hz.

The proposed algorithm is shown to provide an acceptable level of step detection accuracy even for low sampling rates. The complexity of the proposed algorithm is below that of the conventional peak detection algorithm. There is a trade-off between the power consumption and the accuracy of step detection under the control of the sampling rate of acceleration when considering smartphone usage environments or types of applications.

### 5.5. Limitation and Further Study

In real smartphone usage environments, users may change their device poses while they are in static condition without walking or running. For the proposed algorithm, this kind of device pose change in static condition is not considered, but only the device pose change during walking or running. Therefore, device pose change in static condition may incur peaks and valleys in the magnitude of acceleration, which may cause the wrong step detection in the proposed algorithm. To consider the device pose change in the static condition, walking detection should be integrated into the proposed step detection algorithm, which is left for further study.

## 6. Conclusions

A novel step detection algorithm was proposed for robust step detection in real smartphone usage environments in which step mode and device pose are continuously changing. The dynamics of smartphones were decoupled into the peak-valley relationship without estimating step mode or device pose. All of the variabilities in the magnitude of acceleration in each step mode and device pose were integrated into stable measures reflecting the short-term and long-term variation of such factors as step average, step deviation and the statistics of time intervals between peaks or valleys. These measures were used for the calculation of adaptive thresholds to validate peak and valley candidates in magnitude and temporal directions. The proposed algorithm was shown to achieve low power operation and an acceptable level of accuracy irrespective of the step mode and device pose; its superiority over state-of-the-art algorithms can be clearly seen. The proposed algorithm can be used for various applications that are based on step detection and that require high precision or low power operation.
